# Hunches that matter: the role of intuitive concern in medical understanding

**DOI:** 10.3389/fpsyg.2025.1508138

**Published:** 2025-07-02

**Authors:** Kerrin Artemis Jacobs

**Affiliations:** Department of Psychology, Center for First-Person Research, Faculty of Health, University of Witten/Herdecke, Witten, Germany

**Keywords:** intuition, concern, medical professionalism, predictiveness, medical understanding

## Abstract

This conceptual analysis examines the role of intuition in medical understanding from a philosophical point of view: (1) Intuition serves an indicative function, whereby it experientially reveals that something is of importance to us, thereby enabling us to adapt and (re)evaluate situations. This results in the emergence of a distinct conative dimension. The intuitive judgments and insights about what matters also come with an urge to act on them, which is crucial for explaining the motivation for proactive prevention of harm and the promotion of well-being. (2) One specific mode of recognizing “what matters” is being *intuitively concerned*. Intuitive concern can be conceptualized as a process that relies on the interplay of intuitive “knowing” and deliberative thinking in clinical decision-making. (3) It can be concluded that these hunches are significant, as they indicate not only what should be taken seriously but also the necessity of achieving accuracy. The intuitive concern is an essential aspect of medical professionalism, both as a reflexive necessity and as an expression of the “art of healing.”

## 1 Introduction—the holy grail of medical ‘understanding’

“(.) Plato’s suggestion that the physician, like the true rhetorician, must take the whole of nature into view remains valid. Just as the latter must draw on true insight to find the right word which would influence those who listen, so too the physician must look beyond the immediate object of knowledge and skill if he is to be a true physician.” Gadamer 1996.

Intuition lies at the core of medicine as a *practical* science (Gadamer, 1996a, p. 31–45). Following Gadamer’s idea of medicine as an *art of healing* (Gadamer, 1977, p. 19; Gadamer, 1996b, p. 103–4), medical professionalism shows in much more than the application of theoretical knowledge: we deal with medicine not only with respect to its technical-scientific aspects but also with a life-worldly dimension, and this must include the many facets of *intuitive understanding*.

For a first preliminary grasp on this notion, it can be stated that intuitive understanding refers to an understanding of one’s own self (e.g., as a medical professional), of others (e.g., as patients, clients, subjects of care), and of the *conditio humana* (“*the equilibrium of life and health*,” cf. Gadamer, 1996a, p. 35–38; Gadamer, 1996b, p. 113–114) beyond the realm of that which is just merely factual in a clinical setting. It points beyond that which can be scientifically described to that which rather must be understood. Intuitions have already been appreciated for closing an important “gap” in medical diagnostics. For instance, it has been suggested to rely on intuitions when applying a general rule to a specific situation of an individual client (see Adler, 2022). This is also compatible with recent claims in the psychiatric methodology where intuitive judgment is discussed as “not inferior to other diagnostic methods” but rather as something that should be “used to suggest, guide, and modify psychiatric diagnosis” (e.g., Srivastava and Grube, 2009, p. 99). This is also confirmed by the methodological reflection of intuition in other medical disciplines, such as expert nursing (as outlined by Benner, 1984; for a critical review of Benner’s influential theory (see Gobet and Chassy, 2008).

Gadamer suggests that healing is always directed toward restoring a balance of life and health and that this requires a *holistic understanding* of medical cases. This view is compatible with a general twofold orientation in medicine: medical professionals not only treat diseases (biological dysfunction) but also preserve and promote health and thus are *concerned* about the well-being of their clients (Jacobs, 2018, p. 284). Medical experts do not limit themselves to a negative concept of health or exclusively to a narrow concept of disease (disease is defined as a harmful dysfunction, and only this is diagnosed and treated); instead, they are oriented toward positive concepts of health in their practice. A positive concept of health complements the general commitment and medical ethical obligation to evidence-based, scientific medicine. Moreover, it is necessary to distinguish between a narrower medical area and a broader area of medical practice in such a way that they each correspond to a specific understanding of health and disease. The narrower or primary area of medical practice is characterized by an orientation toward a negative concept of health and/or a narrow concept of disease. However, this narrower definition of medical practice can be combined with an additional orientation toward positive concepts of health, which capture health through certain theories of well-being. Compatibility can be achieved by understanding the concept of health as an (ideal) complement to a primary orientation toward the goal of remedying dysfunctions. Expanding the view of medical understanding thus can include the consideration of a wider range of pathogenic factors and social constellations as medically relevant (thus objects of *medical judgment*),^1^ e.g., those that may foster the risk for developing a disease or for staying in an unhealthy condition (Küfner et al., 2006). It clearly depends on the design of the diagnostic process and medical intervention possibilities (Bebensee, 2019), but a practical orientation toward a concept of health—toward salutogenesis, not only pathogenesis—in medical diagnosis, prognosis, therapy, or counseling generally contributes to such a holistic understanding in Gadamer’s sense: The *intuitive extension* of medical understanding resonates with what Gadamer has described as a professional skill for “distinguishing between the particular constitution of the organism in question and what is actually compatible with that constitution” so that medical professionals^2^ “must be able to look beyond the ‘case’ they are treating and have regard for the human being as a whole in that person’s particular life situation” (Gadamer, 1996b, p. 42).

Medical professionals also face certain *limitations* “imposed on them by the fact that the fabricated product of their art is detached from its process of origin and given over into the free uses of others,” which “in the case of the doctor […] becomes a genuine case of self-limitation” (Gadamer, 1996a, p. 43). So, all understanding requires medical professionals to “be capable of reflecting on their own medical intervention and its probable effect on the patient. They must know when to stand back. […] The doctor’s art ultimately consists in withdrawing itself and helping to set the other person free” (Gadamer, 1996a, p. 42–43). Karl Jaspers has pointed in the same direction when emphasizing that medical understanding always appeals to freedom (Jaspers, 1950, p. 221–230; 222; transl. KJA). What must be added is that these limitations are not only imposed by an obligation to medical ethical duties (e.g., as outlined in the *Charta of Medical Professionalism* by ABIM Foundation and ACP-ASIM Foundation (2002)—first and foremost of respecting the autonomy of “individuals toward all medical efforts of healing are directed” (Gadamer, 1996a, p. 43)—but appear as *limits of understanding*, *thus also limits of intuiting*, themselves. This stems from the practical insight that the art of healing is, on the one hand, ultimately determined by not being able to medically alter, restore, or produce in medical practice what ultimately only “life itself can bring” (Jaspers, 1954, p. 35). Gadamer also states that at a certain stage in the healing process, the “doctor’s profession [is] possessing in that respect *symbolic value* as certain things are out of his control (cf. Gadamer, 1996c, p. 89).

On the other hand, it is also limited by what Karl Jaspers has addressed with the so-called *incomprehensibility theorem* (Jaspers, 1913): This is indebted to the idea that there are experiences no one can “empathize” with. This means that there is a limit to understanding (another being’s mind) even if one is a medical professional, e.g., a trained clinical psychiatrist or psychotherapist. Although there have been attempts to “clinically objectify” such limitations of understanding, these efforts seem to somehow obscure both the concept of (mental) illness and the idea of medical understanding itself, as it is obvious that medical understanding amounts to more than “mere comprehensibility” and that (in)comprehensibility itself neither contributes to an adequate concept of illness nor does justice to the fact that the respective (illness) experiences are determined decisively by the subjective state of the person affected, i.e., are determined by the client’s own *illness-identity* (Pelters, 2004). One must keep these methodological reflections on limitations of “understanding” in mind, i.e., the respective troubles that come with the idea that medical (mis−/non-)understanding is solely anchored in (in)comprehensibility, which, however, is a critique that all theories must face that proclaim some sort of professional “sensing” skills (e.g., empathy) as the *via regina* to understanding.

It is, however, also clear that the needs of clients are better comprehended if medical professionals develop some “empathetic” skills (Moudatsou et al., 2020). Instead of focusing on empathy, I suggest concentrating more on intuitive competencies and what it means to professionally deal with one’s medical intuitions, respectively. An appreciation for the importance of taking intuitive knowledge more seriously in medical understanding should be fostered and cultivated through ongoing education of healthcare professionals. Moreover, it is worth noting that professionals can be intuitively concerned about a client, even when there are limits to their capacity for empathy. This adds weight to a general critique that argues “the dichotomous conception of clinical empathy (divided into cognitive empathy and affective empathy) may have negative impacts and hinder a more comprehensive, holistic conception of clinical empathy” (Guidi and Traversa, 2021, p. 906). It is worth noting that this may even restrict a more holistic conceptualization of medical understanding. Empathy is certainly an important skill in medical practice, but intuitive concern might be the “thicker” concept needed to address the holistic meaning and broader scope of practical medical understanding. This must be specified: to be intuitively concerned means here not only being concerned about people that are recognized as “clients” or “patients” (for the terminological differences, see Shevell, 2009; Nair, 1998). This has become exemplarily addressed with patient-care approaches that mainly highlight the subjective experience of persons being “treated” as “subjects of medical concern” (see Oben, 2020) and/or elaborate the specific societal dynamics of (distortions) of medical understanding (for this perspective, see Lyon and Mortimer-Jones, 2021; Jacobs, 2012a, p. 79–90). However, medical understanding encompasses, in principle, concern about almost all factors of the medical scenario, e.g., how to address environmental circumstances in a particular medical setting, particularly considering uncertainty, time pressure, or resource limitations, and so on. Moreover, several other important aspects of the role of intuition for medical professionalism are often overlooked, such as intuitions that are not directed toward or experienced in direct social encounters with people but inform as intuitive insights on what scientifically matters, e.g., a new research hypothesis in medicine. In this sense, my analysis may also be productive in working out other facets of intuitive *understanding* through this shift from ‘empathy’ to ‘concern.’

This analysis catches up with the discussion about the (restrictive) handling of the diagnostic scope of medical reasoning in the first place. The scope of medical judgments, and thus also of the interventions to be applied, is limited by strict adherence to the guidelines of evidence-based medicine (as described by Haynes et al., 2002; Sackett et al., 1996). Consequently, one might think that intuitive judgments are ruled out, as they are often characterized as pre-factual knowledge *in the absence of any better knowledge* or factual evidence. Moreover, one might think that it may be hard to use intuitions to justify medical decisions in terms of consensus-oriented medicine, as they are thought of as being notoriously “subjective” and lacking a connection to deliberative thinking or reason. In contrast, my hypothesis is that intuitive judgments are compatible with and contribute to both evidence-based and consensus-based medical praxis. As this analysis is restricted in scope, the goal is to provide reasons to accept that an underestimation of intuitive knowledge as a reliable source and expression of practical medical reasoning stems from a misconception of what underlies intuitive sensing and often misses the whole point of how exactly these hunches “matter.” It must be demonstrated how intuitive concern is informed (often through extensive) expert knowledge about research evidence, which it relies on and cultivates through clinical experience and that its intentionality includes taking patients’ values seriously as well. Intuitive judgments, moreover, become re-appraised in light of the respective guidelines and norms of rationality provided by an evidence-based and consensus-based framework, while a consensus-based model primarily provides a richer (normative) frame for discussing how intuitive concern is an expression not only of subjective but also of intersubjective (even joint modes of) reasoning.

That being said, what are these “hunches,” and why do they matter?

## 2 “Hunches that matter”—intuitive concern

*“The body knows everything. We know very little. Intuition is the intelligence of the organism.” — Fritz Perls.*

To clarify the role of intuition in medical judgment or, rather, *understanding*, one must provide a concept of intuition. I will focus on intuiting based on “predictive” skills, as predictiveness is seen to be a structural and conceptual (pre-)requirement for almost all concepts related to “sensing” others. Intuitive experiences bring something to the forefront of our consciousness, indicating that something is of (actual) salience. Intuitive experiences come with the motivation to reassess a situation and to adapt accordingly. As predictive beings, we intuitively register what (might) be of import and thus intuitively sense what is (or even should be) a matter of concern, even in medical situations. I have already sketched this indicative function of intuition (see Jacobs, 2023), but I have further substantiated this idea with the concept of ‘intuitive concern’ as a motivating (proactive) mode of harm-preventing action. This is a concept that matters practically, specifically in the realm of medical interventional praxis.

### 2.1 Types of intuition

Intuition is anchored in the basic information processing dynamics that take place “at the fringe of human consciousness” (Zander et al., 2016, p. 3; see also Mangan, 1993; Norman et al., 2006). *Intuitive judgment* has been exemplarily described as a “subjective experience of a mostly nonconscious process that is fast, a-logical, and inaccessible to consciousness that, dependent on exposure to the domain or problem space, is capable of accurately extracting probabilistic contingencies” (Lieberman, 2000, p. 110–111),^3^ and also has been differentiated from the notion of *intuitive insight*, the capacity associated with gaining an accurate understanding of something, thus being a base for the development of a new hypothesis and perspective on a particular case (this has been described already as the “creative function of intuition” by Polanyi (2012).

The main idea has been that intuition must be somehow conceptualized as *distinct* from higher cognitive decision-making processing (e.g., Hogarth, 2001; Dane and Pratt, 2007). The so-called “dual process theories” (Epstein, 2010; Evans and Stanovich, 2013) and, respectively, “two-system-framework” models (e.g., Tversky and Kahneman, 1983) have excessively stressed the difference between fast (intuitive) and slow (deliberative, analytic) thinking (e.g., Stanovich and West, 2000; Kahneman, 2011). In the meantime, these models that assume two *completely separate* systems have been criticized (for instance, by Keren and Schul, 2009), and there is generally also a growing interest in integrative framework perspectives that consolidate and combine various approaches toward intuitive decision-making (e.g., Launer and Çetin, 2025). Although it has been objected that dual theories are too narrow and are (still) only of little scientific advances, as there is a lack of conceptual definitions and stringent criteria for testing the empirical evidence for two-system theories, this “gap” is still active: This has been the theoretical ground on which a dichotomy between “quick intuition” (e.g., in moral psychology as “*fast and frugal heuristics*” (Gigerenzer, 2008) vs. in-depth reasoning or “deliberate thinking” has been instantiated. Intuition has been described as “the ability to understand immediately without conscious reasoning (…) the *rightness or wrongness* of a person, place, situation, temporal episode or object” (McCrea, 2010, p. 1; italics KJ). It exemplarily stands for different pathways toward “rightness and wrongness.” It has led to a differentiation of neurobiological mechanisms correlating with different forms of judgment (for instance, in the influential research on moral dilemmas, as conducted, e.g., by Greene, 2013). It can, however, be stated that this analysis assumes that—despite all differences in what appears subjectively as intuitively striking to someone—intuitive judgments can themselves become objects of reflection in deliberative processes. So, instead of following a “single-process-view” provided by *theories of judgment—*that can be subdivided into one strand that states that *reasoning* dominates judgments (which makes sense also with respect to a developmental perspective, e.g., Piaget, 1965), in contrast to the other strand that postulates that *intuition* is the dominating process (or “comes first”)—it is assumed that since all deliberative processes necessarily are already pre-informed and emerge from an *interplay* of (pre)reflexive and (self-)reflexive evaluative dynamics that *together* shape our reasoning processes, to generally dissect intuition from reasoning or to ask “what comes first” makes little sense. Although intuitions cannot be intentionally evoked (e.g., Topolinski and Strack, 2008) intuitive judgments have been suggested as already *pre-determining our deliberation processes* (cf. Dörfler and Ackermann, 2012, p. 556–557) *and* as being constitutive for having insights (according to a *continuum perspective of intuition* (see Zander et al., 2016, p. 2–3). So, intuitive judgments feed into “the incubation stage of insight where rich informational chunks are restructured into new understandings” (McCrea, 2010, p. 30). This has led to the hypothesis of considering both concepts “more sister cousins than orthogonal constructs” (McCrea, 2010, p. 30). Both types of intuitive “knowledge” are required in medical reasoning: The aspect of immediateness is central for intuitive judgment and is important in scenarios that come with greater uncertainty and that require fast reactions (for the differentiation of three heuristics that are employed in making judgments under uncertainty, see Amos Tversky and Kahneman, 1974), while intuitive insight might help to find (even new) solutions. It has innovative potential with respect to both “thinking *inside and outside* the box”: it is a cognitive process that brings about novel ideas or solutions, which, however, all require “inside-the-box-thinking” (Weisberg, 2009) as it already presupposes (an often extensive) knowledge in a particular area. This now gets further substantiated below with respect to an acquired “tacit background knowledge” that allows us to specify how intuition already pre-informs deliberative processes and thereby “transcend[s] the capacity of merely intellectual methods and the techniques of discriminating the factors of the situation” (Barnard, 1938, p. 235) quoted by Dörfler and Ackermann 2012, p. 555), thus reminding us of Gadamer’s idea of looking beyond the immediate object in medical reasoning.

### 2.2 Intuition as predictive sensing

In the process of intuiting, we transform a knowing-that (i.e., a theoretical “implicit” knowledge) into a practical knowing-how, which exemplarily expresses itself in our immediate awareness of what *matters* in a specific medical case. This has been explored for its correlation with successful medical practice, particularly in good performance in clinical decision-making, especially in clinical settings that require quick intervention and are shaped by greater uncertainty (Hall, 2002). In further asking how intuition coins medical “expertise,” it has been suggested that we consider two approaches to intuition and expertise, namely heuristics and biases on the one hand and “classic” decision-making on the other, to find out what “really” underlies professional intuition skills, i.e., to separate “true intuitive skill” from mere overconfident and biased impressions of medical professionals (see Colter and Mills, 2020). It has been shown that the *quality* of an intuitive judgment depends on an assessment of the *predictability* of the environment, as well as the individual’s opportunity *to adjust* to the respective environmental conditions and regularities. Predictive *skills* are key to “intuitive” clinical reasoning, more precisely, to “clinical judgment” (to use that notion following the critique of the use of intuition in medicine provided by Feinstein (1967).

Intuition must not be tied to one faculty (e.g., cognition) of the mind but can be recognized for its *multi-potential aspects* (Dörfler and Szendrey, 2008, cited in Dörfler and Ackermann, 2012, p. 549). Consequently, the intuitive experience must not exclusively be discussed in reference to “knowledge” in terms of beliefs but must be addressed with respect to a plurality of evaluative content that impregnates “having an intuition.” This multi-potentiality can be embraced by conceptualizing intuition as a basic mode of *evaluation* that takes place at the *interface of pre-conscious appraisal and conscious assessment*, as, for instance, the *predictive processing framework hypothesis* suggests (cf. Miller et al., 2022). The idea is that we adapt to current situations, namely by constantly comparing incoming sensory information and current experiences against the “stored” knowledge and memories of previous experiences. Following this idea, a mismatch (something not predicted) leads to an update of cognitive models (the default mode) through the constant, ongoing dynamic of adjusting between prior modules and the information of current experiences. This has been described as a fast, effortless (Hogarth, 2001), automatic, and subconscious process, which amounts to the typical intuitive experience of “knowing that, without knowing why” (Claxton, 1998, p. 217). The prototypical conscious experience of “having an intuition” can thus be seen as the result of having already detected something (unconsciously/“pre-reflexively”) without (yet!) having (consciously/reflexively) registered it. It has been suggested that the ‘error signals’ (“mismatches”) determine “whether the model is either amended and its current hypotheses are changed to accommodate the mismatch (‘passive inference’, perception), or the hypotheses are kept fixed and lead to resampling of the sensory states according to the current model (‘active inference’, action)” (cf. Miller et al., 2022, p. 798; see also cf. Jacobs, 2023, p. 5). This is also compatible with classic psychoanalytic descriptions: to “*become conscious,*” this information must be *pre-consciously* available. Conceptually, an intermediate level can be assumed as a bridge between the “unconscious” and the “conscious” sphere so that intuitive experience is one phenomenon of a “breaking” through of registered (and stored) information that a person has not been currently aware of but which can be retrieved (see Larsen and Buss, 2013). This interplay of a “tacit” background (knowledge) and our specific (“foreground”) encounters implies that intuitive experience is never fully independent from the acquired (habituated) pre-reflexive evaluative patterns, and thus our “*tacit knowledge*” (e.g., Bowers et al., 1990; for a detailed description of the *tacit system,* see Hogarth, 2001, p. 191ff). Crucial for ongoing *adjusting dynamics* (or to allude to Gadamer’s notion, the “equilibrium of mind”) between prior models and the information of current experiences is that it provides us with *relevant information for reassessment*: Intuitive experiences allow us to adapt to a current situation based on registered changes provided by information processing of the predictive network. This means that *intuition is an adaptive skill of the predictive mind*, a concept also discussed in the context of a narrower evolutionary psychological view (e.g., Cosmides and Tooby, 1994). This also indicates that our predictive sensing may become more and more “reliable” as more “background” information is acquired. If we follow this heuristic approach of “sense-making,” intuitive perception of self, others, and the world does not take place in the absence of any “better knowledge” but is a “basic” appraisal of one’s actual situation against the evaluative (subconscious and pre-conscious) background of memorized experiences and knowledge. It is with respect to the ongoing looping dynamics of evaluative (re)appraisal that these implicit knowledge structures are the result of all previous deliberative enaction but simultaneously also the actual point of reference for enaction, which minimizes the conceptual “gap” between intuition and reason. The default mode of all conscious evaluative self-and-world-orientation is constantly updated, for instance, through learning from (repeating) scenarios that share some prototypical features, which can be discriminated, recognized, and remembered due to perceptual pattern recognition (see, for instance, Hodgkinson et al., 2008).

Intuitions rely on *knowledge structures* (e.g., Bruner, 1960, p. 57), and here particularly, the differences in implicit memory functions have been further explored (e.g., Volz and Zander, 2014) who have suggested that intuition differs, e.g., from *priming* in terms of the format in which information is stored in memory, as well as in the signal that accompanies the cognitive process, respectively). This is the reason to assume that *qua* being experienced (or skilled) in something, one simultaneously develops a fine-grained sense that allows one to detect even subtle changes (registered as inconsistencies/“mismatches” or as consistencies/“matches”) against a background of already incorporated (embodied) skills and knowledge (Jacobs, 2013, p. 2, 3). This is relevant for explaining, for example, how medical professionals develop and can even train or cultivate their intuitive skills and why the quality of intuitive judgments might inherently depend on the scope of implicit knowledge one has acquired through practice. This knowledge is, on the one hand, subjectively unique due to different experiences, but on the other hand, it includes (specific) knowledge and skills that are intersubjectively shared and acquired by being part of a certain culture, community, group and so on so that we all share (to a certain extent) an implicit “*intuitive horizon*” of knowledge together. In this sense, it can be assumed that we share a common ground with others and rely on it in our intersubjective exchanges. Even broader categories, such as “common sense” theories of morality or rationality, can be referred to as those normative frameworks that “predictive” minds are embedded in and rely on and that become re(actualized) and maintained in and through modes of intersubjective relatedness, respectively. This points toward intuiting not only as a subjective evaluative mode of self and world experience but also to the *inter*subjective dimension of being intuitively related to others. In this sense, our personal “predictive” space already includes the other, and we, respectively, intuitively adjust toward and synchronize our behavior (together) with others, particularly as intuitive sensing allows us to *register or detect a great* var*iety of changes.* Being intuitively related, particularly in modes of immediate interaction with others, is moreover central for developing a sense of self and a sense of others and the world, which points to intuition as a primordial mode of and as an experiential necessity for social (re)cognition. Consequently, predictiveness can be reemphasized as a structural pre-requirement for experiencing oneself in meaningful relatedness to others and the world and for also developing an explicit knowledge about self, others, and the world [which can be further outlined, e.g., with respect to developmental-psychological dynamics of intuitive inter-affective relatedness, e.g., as described by Stern (1985) or Fuchs (2011), p. 209]. Another way to address this is possible with Ratcliffe (2008) notion of an experiential, *affective* background that shapes our sense of reality, normalcy, and belonging. Such “feelings of being” are assumed to be the affective background structure according to which one experiences self, others, and the world as somehow meaningfully related (Jacobs, 2012b, p. 143–144). Under the auspices of such an implicit affective “background” structure, one could exemplarily elaborate how specific changes of “existential feelings” (e.g., our sense of basic trust and belonging) correlate with significantly altered predictive processing dynamics: Particular intuitive experiences (e.g., “gut” feelings) then may be already indicative of changes in the affective background structure that shapes meaningful relatedness to self, others, and the world.

This points to both the relevance of (distortions) of predictive sensing for the description of psychopathological phenomena (e.g., for the case of paranoia, see Jacobs, 2023, p. 2), but most importantly, its relevance for describing “flawed” medical judgments: they can become flawed by (cognitive, affective, conative, thus: evaluative) biases, which can be traced back to *biased predictive sensing*. Consequently, it cannot be generally assumed that subjective, intuitive experience is always a reliable indicator for accuracy (e.g., of a medical judgment), and this requires that medical professionalism stress the respective self-reflexive skills to be able to re-assess, i.e., to question one’s (cognitive, affective, conative, or generally evaluative) biases and to actively “*de*biase” one’s thinking (Corrao and Argano, 2022; Vela et al., 2022). This has been discussed as an important strategy for preventing “deprofessionalization” (Siepmann and Groneberg, 2012) so that a medical *déformation professionnelle* is explained under the auspices of *predictive biases.* However, far from being a happenstance phenomenon or simply a matter of “luck”, correct predictive sensing is the signature of medical professionalism, which leads now to the question: What means *intuitive concern* as an immediately *knowing* that something is a matter of import, thus an experience that should be taken seriously, and moreover can motivate (pro-social) action?

### 2.3 The indicative function and motivational dimension of intuiting

Intuitions always reveal the (actual) mode of our meaningful relatedness (to self, other, and the world) and allow us to adapt because they *indicate that something is of (actual) salience*, which has been sketched above as being always already pre-informed by implicit knowledge structures (Jacobs, 2023, p. 5). Intuitions signal to us what is or should be taken seriously and, thus, what might be a matter of concern. Granted, one can be intuitively concerned about almost everything, as everything can become a matter of import, appear as somehow meaningful, or lose its significance for us, respectively. It is not nitpicking to ask what exactly is meant by intuitive concern.

From a *phenomenological perspective,* one could say that central to the experience of *intuitive concern* is that there has already been some sort of change of meaningful relatedness toward self, others, and the world. One could speculate that depending on how an intuitive experience expresses itself—think of the intensity of an urge to “follow our guts”—the feeling of certainty that something must be the case despite a lack of evidence (yet!), or having a “eureka!” moment—a moment typical for intuitive insights. These experiences indicate *how much* something “matters,” which points particularly to the “felt” as well as the explicit affective aspects, as well as in the (often overlooked) conative momentum of intuitive experiences, i.e., their *motivational impact*. Alternatively, from a *conceptual, analytical* view, we can try to dissect the actual phenomenological experience of “being intuitively concerned” from that underlying mechanism that “activates” or “primes” it, according to which the “indicative” role would be reserved for the dynamics of the predictive system network (the signaling function). Accordingly, intuitive concern is that which correlates with—or is the conscious experience of—changes in salience or import, emerging from the dynamics of the respective predictive system. Nota bene: intuitive concern does not necessarily imply being worried about something. However, this is a good example to illustrate that this conscious mode of worry is already an indication that something has been perceived as being important. So, the worry “is” the experience of a (change) of meaningful relatedness, and simultaneously, being in this state implies the intuitive judgment or insight that there is something to be concerned about, based on the anticipation that some kind of “good” might be negatively affected. It has already been mentioned that listening to our “*holistic hunches*” or “*gut feelings*” (as Bechara and Damasio, 2005 have coined it) does not guarantee that things are actually really as we predict them; thus, it might occasionally also lead to a sort of suboptimal or even maladaptive behavior if we “just” follow our intuitions (“this urge”). However, the idea of intuition as already referring to pretty solid structures of acquired and shared knowledge and skills, which therein are stable (normally: not pathologically rigid, e.g., overly biased) experimental background structures, points toward those hunches in which we sense “that something is not right,” in which we experience feelings of doubt, unease, suspiciousness, and so on. These should not be underestimated as signals to probably consider a re-assessment of the situations we actually find ourselves in, and probably signals to also adjust our behavior.

Thus, the indicating function of intuitive sensing yields a *motivational* momentum, allowing us not only to intellectually evaluate but also to (en)act on intuitive concern, thereby adapting to a specific situation in a particular way. This gives rise to the idea that intuition is either a precondition or even a specific type of (core) *moral* judgment. Intuitions can be re-assessed in terms of “moral” heuristics, i.e., the processing that takes place against the backdrop of our moral capacities and repertoire of ethical know-how that shape our experiential default mode. Consequently, altered social recognition relations (for which moral or ethical conflicts are paradigmatic) can be explained in terms of (a-)typical or even (dys-)functional predictive dynamics (thus, intuitive capacities). Jonathan Haidt (who coined the term “*moral intuition,*” see Haidt, 2001). For a critique, see Saltzstein and Kasachkoff (2004) has stated in his explanation of what shapes moral guidelines (Haidt, 2003) the importance of emotional experiences for understanding the capacity for judgment (in moral reasoning), thus stressing particularly the affective experiential dimension of “*intuition*” and its role in moral understanding. Another approach to immediate knowledge (of “right and wrong”) is Shaun Nichols’s neo-sentimentalist approach, in which he suggests an affective mechanism that is spontaneously activated “in the conspicuous absence of any judgment that a transgression (or even an action) has occurred” (Nichols, 2004, p. 63; see also Nichols, 2002). Nichols appears to be targeting a specific type of *concern* that plays a decisive role in our perception of certain norm violations as “wrong” in a distinctly different manner than other norm violations. This basic ability to resonate or “minimally” ascribe suffering to other persons is tied to *affect* in this account of (core) moral judgment. Indeed, it would make sense to consider predictive sensing as the relevant underlying mechanism for *moral concern* and/or intuition as pre-informing moral judgment, as we are already intuitively “alerted”—for instance, when (possible) transgressions of harm norms are predicted, thus initiating the anticipation of a need for readjustment. These are normally where social interrelatedness or environments can be judged to be “concerning” with respect to what intersubjectively could be agreed upon as (potentially) non-trivially harmful, i.e., where “objective” or universally valid norms (e.g., of physical or mental well-being) are at stake, respectively.

Against this backdrop, and particularly in reminding us of the tacit knowledge structures we share, intuition is significant, as it enables us to discern whether something is of import, *particularly* with respect to those basic (universal) norms and conventional rules that regulate social recognition relations. We can immediately register and (ex-post refer to) “red flags,” i.e., particularly those situational cues that—depending on our specific default mode and implicit knowledge—are registered and evaluated as alarming. This normally primes intuitive experience that we cannot simply ignore, or, if we are trying to do so, requires a lot of effort—a higher-order volition—to “rationalize” our guts away. Thus, intuition can have this explicit moral or ethical relevance of registering what is (or might be) a matter of concern. These experiences may also vary, ranging from one point of the spectrum, which we may call “intuitive overconfidence,” referring to those experiences of immediately “knowing” for certain what is “good” or “bad,” which may come with a respective stronger urge to follow this intuitive assessment, to more moderate or mild intuitive experiences of concernment where we anticipate that certain things should be (probably) taken (more) seriously but correspond with less of a “drive” to put this knowledge into action. It relates probably to those situations in which we are trying to find out whether that which is predicted can be further evidenced and might be accompanied by ambivalent feelings toward one’s concern. The other end of the spectrum could be reserved for those states of diminished intuitive concern that might get easily ignored, for instance, in light of preoccupying foreground experiences that dominate one’s situation in such a way that one is not overly concerned *about* one’s intuitive concern. As predictiveness is never independent of the specific situation someone is in, it may not only depend on a person’s intuitive (in)sensitivity (i.e., introspective capabilities) or individual sensorimotor coupling capacities (such as individual responsivity or resonance capacity) of a person but simply on the particular situational conditions in which intuitive concern is only minimally or not activated at all. This does not rule out the possibility that one *should* have been intuitively concerned or retrospectively wonders why one did not anticipate in the first place that something would be very likely to happen or would be the case. So, perceptual biases determine whether our predictions are correct or not (i.e., whether the content of what has been perceived as a matter of import holds factual ground), but one can also be biased with respect to the correct assessment of intuitive concerns. In contrast, intuitive concern can also “go wild”: then we experience things of importance or particular concern, also in terms of explicit worries, where there is actually no evidence or good reason for anticipating harms and threats. This points to the negative effects of significantly altered intuitive access to the self-world and to predictive errors that matter *because* they initiate actions that can lead to experiences of (non-trivial) harm. The intuitive concern is a vital source of knowledge since it provides us with some sort of sense of or about a “good,” particularly in indicating that something might be a matter of concern but can also be an inevitable source of failure. Although it may come with the potential to be a misguided, intuitive *concern*, in principle, it can be seen as an *enabling* action due to the priming effects that result from registering some alterations in meaningful relatedness. It is precisely the ideas of harm avoidance and well-being associated with them that trigger “intuitive concern.” “Sensing others” may prosocially motivate, implying that we should not underestimate the conative aspects of intuition, which are tied to the indicative function of intuitive concern. This might offer an alternative way to explain what motivates people to be concerned about others (well-being). It is also clear that “understanding” includes much more than concernment, but in principle, ‘intuitive concern’ might be a prototype of (basic) “moral sense(ing),” particularly if we stress “intuiting” in terms of predicting potential harm or transgression of those norms that indicate impairment of well-being and prime someone to adapt—i.e., to react—which can include harm avoidance but also allows for (proactive), prosocial behavioral adjustment, respectively. It has been outlined that predictive skills are a structural pre-requirement for social (re)cognition. Thus, it is also a good candidate for the description of prosocial motivation, including proactive engagement due to the anticipation of (potential) harm or impairments of well-being. Phenomenologically, this refers to the fact that we do not only just “passively” register what is/might be of import but often, indeed, are motivated to follow these “hunches” we experience, believing that they (must) somehow matter. My analysis can only hint at this (still poorly discussed; pre-)*conative* aspect of intuiting, but it can at least mark the crucial point of departure for a future, more detailed explanation of (pro)social motivation considering predictive sensing (Jacobs, 2024). To paraphrase Frankfurt (2006): The notion of intuitive concern allows us to explain what “*taking things seriously*” underlies (i.e., predictive sensing that indicates that something is of import and/or matters) and how this is connected to “*getting it right*.” The ladder can take many different forms, but it is rooted in an intuitive concern for oneself, others, and the world. Intuitive concern is never a “solo show” if one keeps the idea of the “shared intuitive horizons” in mind: the other is part of my predictive space, and this allows for the intentional scope of intuitive concern not to be restricted only to myself, but to extend to others and the environment. The motivational momentum and explicitly proactive and/or even prosocial motivation might be primed particularly by registering (potential) harm. It is risk assessment against the backdrop of our “tacit” or implicit knowledge. This connects my analysis of intuitive concern to theories of empathy, but only insofar as these theories could agree on predictiveness as the underlying, most relevant aspect for proactive and/or prosocial motivation. This leads me to provide a final example of what “getting it right” can mean, namely, addressing one’s intuitive concerns in a professional manner. This is considered a way of “being concerned or caring” about one’s intuitive concerns, simply because significant harms can also be prevented when predictive errors are minimized, particularly when other people’s well-being might be directly affected by one’s intuitive judgments, as is most likely to be the case in medical decision-making:

## 3 Taking hunches seriously to get it right

*“The path of reflection is the path of compromise.” Agustina Bessa-Luís.*

Instead of describing how intuitive concern becomes “utilized” in special treatment settings (such as psychotherapy), I focus on its specific role in *medical decision-making*. One can ask, what is needed from the intuitive medical professional to *properly* handle intuitive concern (so to speak, with care) in processes of medical understanding? Professional handling of intuitive concern can be pinpointed with Gadamer’s description of *mastering one’s skill*: “One will find out in (…) what one calls general ‘practice’ that the more one ‘masters’ one’s know-how, the more one possesses freedom vis-à-vis this know-how” (Gadamer, 1996b, p. 21–22). In a nutshell, we must place intuitions in the picture of medical reasoning where they belong, and this implies that reasoning is *not literally out of intuitive reach*, particularly if we do not conflate *medical understanding* with quick and intuitive evaluation. Rather, it is a compromise between intuition and rational consideration in medical judgment, which Gadamer addresses with the ability for professional self-reflection. Despite the difficulties that come with the notions of “reflection” and “self-reflective practice” in medical professionalism, these have been equated to a wide spectrum of activities that have inspired attempts to come to a unified definition (e.g., as suggested by Mantzourani et al., 2019), who provided a five-component model of reflection that takes attentive, critical, exploratory, and iterative thinking into account as an underlying conceptual framework to explain what facilitates reflection of professional practice). With respect to what is needed to *place intuitions where they belong,* one can be reminded of that *(self-)reflexive process* of professional distancing. On the one hand, it allows one to “rationalize” intuitive concern in light of reason that is informed by the rationality of empirical-evidence medicine, and on the other hand, it can appreciate the epistemological value of intuitive concerns in its particular function to provide additional insight to one’s purely factual, empirically based assessment of a medical case (e.g., such as reflections on the life situation of a client as a whole, which I have elsewhere addressed, e.g., Jacobs, 2022). It appears that the more experienced physicians are, the more likely they are to assess for themselves whether they can trust their intuition. However, relying on experience is only half the battle. The other approach is to remain open to the entire process of re-evaluating the specific content of intuitions, i.e., finding a proper way to align them with what is considered rational. The best-case scenario would be an alignment between *guts and ratio* that might be best put with the idea of “soundness”: That what we already have assessed as most reasonable is “confirmed” by intuition (which would allow us to claim not only an indicative but also an assertive function of intuitions), and vice versa, so that there is no tension between what (inter-) subjectively is held to be rational and what we (intuitively) feel, desire and think about a medical case. Granted, dealing with intuitions professionally implies letting go of the idea that one can always flawlessly bridge the worlds of intuition with that which “makes the most sense” regarding empirical evidence and theoretical knowledge. As a *practical* science, medical understanding requires professionals not only to make evidence-based judgments but also to exercise care in their intuitive judgments. Finding the right diagnosis and treatment requires us to be able to justify why we believe we should trust our instincts or not. This is, as already mentioned, often inspired by a desire not only to falsify or test intuitions by attempting to achieve a better understanding of “what is really going on,” but is also motivated by the practical necessity to decide what is, above all, considered reasonable. “Reasonability” can refer to both a subjective, rather idiosyncratic way of making sense of one’s intuitions and to an intersubjectively consensually agreeable (rational) way of deliberation and action in medicine. Apparently, we cannot simply “rationalize our guts away,” but we can, indeed, rationalize them. This does not necessarily imply that reappraisal of intuitive judgments somehow weakens their (motivational) impact, but rather that they fully reveal their import in the very process of assessing what is literally concerning with respect to a holistic view of the medical situation. Taming intuitions in light of (reason)ability does not necessarily imply diminishing their motivational momentum or treating them as merely a “felt” add-on to rational decision-making. Rather, it means appreciating them in the fullest sense as an expression of knowledge about what (really) matters, especially with respect to socially embedded conditions of a specific case.

This requires a kind of reappraisal of intuition, which involves claims for making *transparent* how intuitive judgments and insights are perceived. For instance, an idea may be considered far-fetched or very likely to be the case given the facts, i.e., empirical evidence and statistical heuristics, as well as one’s professional experience. Here, an additional role might also be that, since intuitions can serve to (re-)assert as well as to question oneself in processes of medical decision-making, it may not only be the “gut” (grains to the rational mills of decision-making) but also valuable for creating this startled moment and doubt that allows one to question one’s purely empirically-based assessment about a medical case. This can also become relevant when several people try to determine what would be medically the best course of action, especially when medical professionals share the same intuitions about a specific medical case. While the proof always lies in the pudding, if others share our intuitive concerns, this might be one additional motivational factor for “taking some risks” in favor of intuitive judgments and insight. This includes, as mentioned above, acknowledging that each “profession” yields some risks of a *déformation professionnelle*, i.e., a habitually acquired set of evaluative patterns that impregnate one’s self-understanding as a professional and potentially limit awareness of a certain rigidity in said evaluative pattern for one’s praxis. Consequently, it becomes even more important to articulate the pros and cons that can arise from following one’s intuitive concern, especially if one’s intuition is literally “counter”-intuitive to the standard procedures of diagnostics and treatment. It may even be assumed that medical professionals act imprudently, if not even irrationally, in “blinding intuitions out,” especially if following or refraining from taking one’s intuitive concern seriously would make a significant difference in diagnosis and treatment and thus most likely have different effects on the patient’s well-being. Here, it is necessary to assess (even conflicting) intuition on a specific case and to calculate the costs and benefits that come with following intuition or not, i.e., estimating the costs of intuitive compliance or opportunistic costs that come with “silencing” or following one’s gut. Even if the appearance of intuitive concern normally is an indicator of something we ought not to transmute, it is less about following or not following intuitive judgments and more about being able to provide reasons why we think that we are doing the right thing in being guided by them or not. It is consistency and *coherence* with intersubjective consensual standards of rational practice that justifies their status in medical understanding. Thus, “being somehow sound with one’s intuition” is apparently not enough of a justification for intuition-based action, at least not from the standpoint of intersubjectively consensual rational practice in medicine. As medical decisions directly interfere with other people’s lives, sometimes including interventions that might have severe consequences for someone else’s well-being, acting upon intuition requires justification beyond a mere intra-subjective standpoint but must be justified in broader terms, consistent with empirical-based medical and ethical standards. Then, the intrapersonal dimension of intuitive medical experience must be bridged with the intersubjective dimension of justification of medical judgments. It is suggested that this requires finding a compromise between a purely empirically based assessment of a medical case and a holistic perception, informed by intuition. This implies the use of discernment for a possible misbalance (between these two forms of judgments) with respect to negative consequences for the overall medical goal of health improvement (which can be generally described in terms of an “overuse” and “underuse” of rationality, see Djulbegovic et al., 2018, p. 655; see also Elshaug et al., 2017).

Medical actions and beliefs are justifiable and thus count as rational when they are informed by evidence. The consistency of one’s judgments with high-quality, evidence-based results is seen as contributing to a proper estimation of specific benefits or harms, which guarantees, in some way, “good” medical practice. The “compromise” view, however, reminds us that neither intuitive judgments nor rationality-based judgments guarantee that the respective medical decision is error-free and that it can lead to suboptimal or even unsuccessful goal achievement. Additionally, medical decisions must also be supported by *reasons*. These reasons may also be provided by intuitive judgments and insights. Consequently, the “best” decisions (given the aim to avoid the negative impacts of overuse or underuse of rationality) would be made in assessing both types of judgments, also with respect to them contributing to the most socially and *normatively* acceptable practice (cf. Mercier and Sperber, 2011). A vis-à-vis freedom of the professional refers to the intellectual stance that understands intuition, as well as an orientation toward empirical facts, can contribute to the rational coherence of medical praxis. This coherence is determined by the right balance of reasons provided by the respective different types of judgment and is defined by the goal of promoting clients’ well-being. This points to the normative dimension of medicine as a practical science, which must not only be rational but reasonable: An intuitive “knowing that” is acquired with growing experience and can be cultivated as contributing to the art of healing, i.e., a “knowing how” that transforms intuition considering that reason(ability) that understands *why* hunches matter. Taking intuitive concern seriously thus points to the core of professionally “getting it right” in medical understanding.

## 4 Conclusion

The objective of this analysis is to present a rationale for the proposition that the “holy grail of medical understanding” can be found in intuition. Intuition plays a pivotal role in medical diagnosis and treatment. Neither of these processes can be reduced to the application of theoretical medical knowledge. Furthermore, the holistic understanding that guides them cannot be explained solely in terms of empathy. It has been proposed that predictive sensing represents a fundamental structural necessity for the intuitive process. This has been motivated by a methodological concern with two prominent gaps: the one between intuition and reason and the one between sensing others and being motivated to act. With respect to the former, it has been demonstrated that the gap can be reduced by emphasizing the inextricable intertwining (ongoing looping dynamics and respective re-appraisal dynamics) that occur during the process of intuiting, which involves a combination of pre-reflexive evaluation and reflexive assessment. The latter has been addressed by emphasizing that predictiveness, and thus intuition, is driven by an inherent motivation to adapt as we perceive and anticipate potential concerns. This has been outlined in terms of registration of harm or transgression of harm norms, with the objective of reemphasizing that, in intuitive concern, something is indicated that is of significant consequence. Further studies will provide additional insight into the specific motivational aspect of intuition. In the context of medicine, it can be stated that intuitive concern is practically significant because it represents an indication of what has been registered as potentially beneficial for the rational goals of harm prevention and health promotion. This implies the provision of reasons—or justifications—for how intuitions are actually consistent with specific medical goals. This is one way to test their coherence with the intersubjectively consensual standards of medical reason. It has been suggested that being professionally concerned about intuition does not mean to “rationalize intuition away” but rather to weigh these perceptions against what is reasonable, *all things* considered. This resonates with what Gadamer has placed at the core of the art of healing: an understanding beyond the merely factually given, in other words, of life itself.

## Data Availability

The original contributions presented in the study are included in the article/supplementary material. Further inquiries can be directed to the corresponding author.

## Footnotes

^1^Medical judgments are *justified* where there is an impairment of very specific functional abilities, since medical action is always primarily aimed at remedying or alleviating physiological and psychological dysfunction. However, medical judgments often entail an additional assessment of said dysfunctions for their (harmful) effects on those (cap)abilities that are considered necessary for a good life, respectively (Jacobs, 2012a; Jacobs, 2012b, p. 79ff., 144–148). The central question then is, of course, how intuitions—intuitive judgments and insight—impregnate that very process of justification.

^2^I will use the notion ‘medical professionals’ to address medical and health-care specialists that are dealing with the biopsychosocial conditions of clients, such as physicians, nurses, psychologists, counselors, etc.

^3^It must be mentioned that specific to Lieberman’s approach is that a sudden insight “into” something is based in nonconscious processes but appears *not* in the form of a *judgment*, as it is in the case of intuitive knowledge. In contrast, *alternative approaches* rather speak of intuitive insight and intuitive judgment as *two cases of intuition* (e.g., Dörfler and Ackermann, 2012).
